# Parasite Richness and Host Condition in *Caranx caballus* (Green Jack): Insights from Artisanal Fisheries of the Eastern Tropical Pacific

**DOI:** 10.3390/ani16081192

**Published:** 2026-04-14

**Authors:** Diego Fernando Córdoba-Rojas, Alan Giraldo

**Affiliations:** 1 Programa Académico de Maestría en Ciencias Biología, Facultad de Ciencias Naturales y Exactas, Universidad del Valle, Cali 760042, Colombia; diego.cordoba.rojas@correounivalle.edu.co; 2 Grupo de Investigación en Ciencias Oceanográficas, Departamento de Biología, Facultad de Ciencias Naturales y Exactas, Universidad del Valle, Cali 760042, Colombia

**Keywords:** monogeneans, digeneans, parasite ecology, *Allopyragraphorus*, *Caligus*, microcotylidae, *Pseudomazocraes*, *Stephanostomum*, *Lernanthropus*, host–parasite dynamics

## Abstract

Parasites are integral components of marine ecosystems, providing insights into host biology, trophic interactions, and environmental variability. This study presents the first systematic characterization of the metazoan parasite community of the Green Jack (*Caranx caballus*) in the northern Colombian Pacific, a region designated as an Exclusive Artisanal Fishing Zone (ZEPA) but with limited parasitological research. Specimens were collected from the Cupica Gulf across wet and dry seasons, and parasitological analyses were conducted to evaluate parasite load, community structure, spatial distribution, and seasonal variation. Of 46 fish examined, 20 were parasitized (overall prevalence: 43.5%), with low infection intensities (1–3 parasites per fish). Nine parasite species were identified, including monogeneans, digeneans, and copepods. Copepods (*Caligus* sp.) represented the most species-rich and dominant group, while *Allopyragraphorus caballeroi* exhibited aggregated distribution. Parasite communities showed low richness and diversity, seasonal stability, and strong trophic linkages to crustacean prey, particularly brachyuran megalopa. Host condition was unaffected by parasitism, suggesting resilience under current infection levels. These findings provide the first reference on parasite richness and diversity for *C. caballus* in Colombia, extending the known distribution of several species within the Eastern Tropical Pacific and underscoring the role of parasites as biological markers for fisheries monitoring and ecosystem change.

## Simple Summary

Parasites are key components of marine ecosystems, providing valuable information on host biology, feeding interactions, and environmental change. This study presents the first systematic assessment of metazoan parasites in the Green Jack (*Caranx caballus*) from the northern Colombian Pacific, a region of exclusive artisanal fishing and limited parasitological research. Across two seasons and three localities, 46 fish were examined, of which, 43.5% were parasitized, generally with low infection intensities (1–3 parasites per fish). Nine parasite species were identified, dominated by copepods (*Caligus* sp.), while *Allopyragraphorus caballeroi* exhibited aggregated distribution. Parasite communities showed low richness and diversity but stable seasonal patterns, which were closely linked to crustacean prey such as brachyuran megalopa. Host condition was not affected by infection, suggesting resilience under current parasite pressures. These findings extend the known distribution of several parasite species within the Eastern Tropical Pacific and highlight their dual role as biological markers for fisheries stock discrimination and sentinels of ecosystem change. Incorporating parasite data into fisheries monitoring frameworks can strengthen sustainability assessments, support food security, and enhance ecosystem resilience in tropical artisanal fisheries.

## 1. Introduction

Marine fishes host diverse parasite assemblages that can affect host physiology, survival, and ecosystem functioning [1,2,3,4]. These impacts may be intensified under climate variability as parasite prevalence and diversity often respond to seasonal and interannual changes [5,6,7,8]. In artisanal fisheries, parasitic infections also compromise product quality and marketability, with implications for food security and human health [9,10]. Beyond pathology, parasites are increasingly recognized as integral components of marine food webs and trophic networks [11,12], offering insights into host diet, resource use, and ecological interactions. Comparative studies in tropical carangids have demonstrated their utility as biological tags for stock discrimination [13,14,15], while broader ecological syntheses highlight their role as sentinels of ecosystem change [4,16,17,18]. Together, these findings establish a conceptual framework in which parasites serve as bioindicators of both fisheries stock structure and ecosystem resilience, situating local artisanal fisheries within global debates on sustainability and climate-driven variability.

Current research increasingly examines parasite biology and ecology to illuminate host dynamics [19,20,21,22], analyze trophic networks [9,11,23,24,25], investigate phylogenetic relationships [26,27], evaluate environmental quality [16,17], predict population trends [13], and project species distributions under climate change scenarios [4,18,28,29,30,31]. These approaches underscore the value of parasites as bioindicators, not only for local stock discrimination, but also for understanding broader ecosystem responses to global environmental change. *Caranx caballus* Günther, 1868 (Carangiformes; Carangidae) is distributed along the northern Colombian Pacific coast and occurs year-round; it is also an important target of artisanal fisheries [32,33]. Given its relevance to coastal fisheries in the central Eastern Tropical Pacific (ETP), recent studies have characterized the *Caranx caballus* fishery in the Las Palmas Archipelago, Panama [34], its feeding habits off Manzanillo, Colima, Mexico [35], and, more recently, its parasite fauna across three Mexican Pacific localities, where select parasite species were proposed as biological markers for stock discrimination [13]. Comparative studies across the ETP therefore provide an opportunity to situate Colombian findings within a regional framework, contributing to a broader understanding of host–parasite interactions and their implications for fisheries management under climate variability.

In Colombia, studies on fish parasite ecology are sporadic and generally limited to a few commercially important Caribbean species. In contrast, the Pacific region—where the largest national fisheries operate—remains understudied. To address these knowledge gaps, this study provides the first systematic characterization of the metazoan parasite community associated with *Caranx caballus* (Green Jack) in the northern Colombian Pacific, within the Eastern Tropical Pacific. We hypothesized that parasite prevalence and mean intensity would vary seasonally in relation to environmental conditions, that endoparasites community composition would be linked to host diet—particularly crustacean prey such as brachyuran megalopa—and that host relative condition (Kn) would remain unaffected under low infection intensities. Our objectives were: (1) to identify the parasite species associated with *C. caballus* captured during artisanal fishing operations; (2) to describe parasite community structure, prevalence, and infection levels across seasons and localities; (3) to evaluate whether parasitism influences host condition under current infection intensities; and (4) to explore the role of parasite assemblages as ecological indicators of trophic interactions, fisheries stock structure, and climate-driven variability. By testing seasonal variation, diet linkages, and host condition alongside the bioindicator role of parasite assemblages, this study presents results from the first systematic investigation of the parasite community associated with an artisanal fish species of importance in the northern Colombian Pacific and contributes to a broader conceptual framework in which parasites inform host ecology, fisheries stock structure, and climate-driven ecosystem variability, thereby linking local artisanal fisheries in the northern Colombian Pacific to global debates on food security and marine resilience.

## 2. Materials and Methods

**Figure 1:**
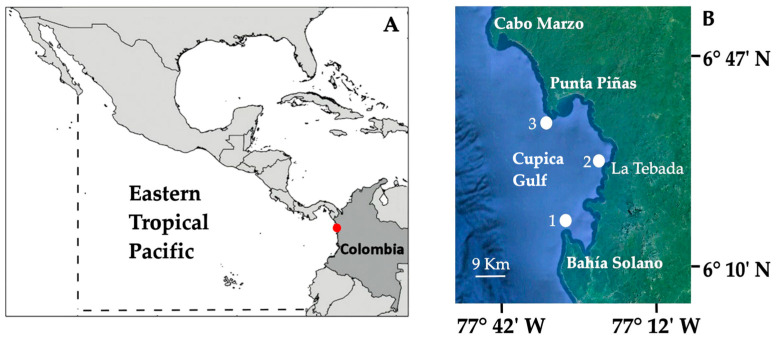
(**A**) Geographic location of the Cupica Gulf (red dot) within the Eastern Tropical Pacific (ETP). The dotted line delineates the extent of the ETP. (**B**) Detailed view of the Cupica Gulf showing the distribution of sampling stations: Bahía Solano (1), La Tebada (2), and Punta Piñas (3). Image: Google EarthR, Landsat/Copernicus, SIO, NOAA, U.S. Navy, NGA, GEBCO. 31 December 2020.

The study was conducted in the northern Colombian Pacific, specifically in the Gulf of Cupica, between Bahía Solano (6°13′38.15″ N; 77°24′15.60″ W) and Punta Piñas (6°39′24.50″ N; 77°31′25.90″ W). This area lies within the Eastern Tropical Pacific, is influenced by the Panama and Colombia Currents, and has been designated as an Exclusive Artisanal Fishing Zone (ZEPA), extending 2.5 nautical miles from Punta Solano to Punta Ardita along the border with Panamá. Three sampling sites were selected within artisanal fishing grounds: Bahía Solano (Site 1), La Tebada (Site 2), and Punta Piñas (Site 3) (Figure 1).

Routine artisanal fishing operations targeting *C. caballus* (Green Jack) were conducted during two contrasting seasonal periods: the dry season (January) and the wet season (April), following regional climatic classifications [36]. Specimens were captured using standardized nocturnal handline fishing (6 h per site). For each fish, standard length (cm) and weight (g) were recorded. External surfaces (skin and fins) were inspected in situ, muscle samples were examined by compression between glass slides, a widely applied technique in fish parasitology [37,38,39,40,41] that is comparable in sensitivity to candling under field conditions, and internal organs were fixed in hot 4% formalin. Gills and fins were stored separately for laboratory analysis. No histopathological evaluation was performed as the scope of this study was limited to parasitological identification, quantification, and epidemiological assessment of infection patterns in *C. caballus*.

Oceanographic variables (temperature and salinity) were measured prior to fishing effort using a YSI Professional Plus multiparameter probe (Xylem Inc., Yellow Springs, OH, USA) following protocols by Giraldo et al. [42], and vertical profile from surface to 80 m depth was registered using a CTD Castaway probe Sontek^®^ (Xylem Inc., Yellow Springs, OH, USA). These parameters were included to explore potential associations between parasite infection metrics and environmental conditions.

Parasite identification was conducted under stereomicroscopy using morphological criteria. Monogeneans and digeneans were stained and dehydrated following the method of Vidal-Martínez et al. [37], while copepods were cleared in glycerin–ethanol solutions [38,43]. Species determination relied on standard taxonomic keys and references [39,40,44,45], and voucher specimens were deposited in institutional collections. Molecular confirmation was not possible, and this limitation has been acknowledged. Epidemiological indices metrics followed those of Bush et al. [41], including prevalence (% infected hosts), mean abundance (parasites per host, including uninfected fish), and mean intensity (parasites per infected host). Parasite communities were analyzed at infracommunity (within individual hosts) and component community (across host subsets and environmental contexts) levels. Community structure was characterized by species richness, dominance (Berger–Parker index), and diversity (Brillouin and Shannon–Wiener indices). Qualitative similarity between assemblages was assessed using the Jaccard index (J), calculated as the proportion of shared species across seasons, and its ecological importance was quantified using the Specific Importance Index (IE = Prevalence + [Mean Abundance × 100]; [46,47]), with parasites classified as primary (>65% prevalence), secondary (40–65%), or satellite (<40%) species [48].

Fish condition was assessed using Fulton’s condition factor, corrected for relative condition [49,50]. Regression parameters were derived from non-parasitized individuals and compared to parasitized fish using *t*-tests in Minitab v16. Stomach contents of *C. caballus* were analyzed to assess dietary preferences and their relationship to parasite load. Prey items were identified to the lowest taxonomic resolution, grouped into categories, and quantified using the vacuity index (VI), frequency of occurrence (FA), and numerical frequency (FN) [51,52]. The Index of Relative Importance (IRI; [53]) was calculated to classify prey groups as low (0–9.9%), secondary (10–40%), or high (40–100%) trophic relevance.

Comparisons of prevalence and abundance among sites, seasons, and host sex were performed using *t*-tests, chi-square (χ^2^), Fisher’s exact test, and Mann–Whitney (MW) U, implemented in Quantitative Parasitology QP v.3.0 [54] and PAST v3.06. Relationships between prevalence and mean intensity were tested using Spearman’s rank correlation (rs). Aggregation patterns were evaluated using dispersion indices (variance-to-mean ratio K, Green’s IG, Morisita’s Im, and Lloyd’s mean crowding IMC) in PASSaGE v2 [48,55]. Diet–parasite linkages were explored by correlating prey categories with infection metrics to evaluate trophic transmission pathways. Statistical significance was set at α = 0.05.

## 3. Results

### 3.1. Host Characteristics and Parasite Detection

A total of 46 *C. caballus* specimens were captured: 18 during the first season (dry, January), all from Punta Piñas, and 28 during the second season (wet, April), of which, 25 were collected at La Tebada and three at Punta Piñas. Sex determination was only possible in the dry season, when gonads were identifiable due to reproductive maturity. Of these individuals, one was immature, 11 were males, and six were females. This information was used to evaluate differences in total length and weight between sexes. During the second season, all fish were either immature or in regression, as gonads were not visually identifiable. Given that all individuals were below the reported mean size at sexual maturity (38.8 cm; [34]), they were considered immature (Table 1). No significant differences in total length were detected between males and females in dry season (MW, U = 26, *p* = 0.48). However, fish captured in dry season were significantly larger than those in wet season (MW, U < 0.01, *p* < 0.01).

### 3.2. Parasite Composition, Infracommunity and Component Community

Parasites were detected in 20 of the 46 *C. caballus* specimens examined, corresponding to an overall prevalence of 43.5%. The Clench species accumulation model indicated that the data fit the expected curve (a = 0.71, b = 0.06, R^2^ = 99.7%), with a sampling representativity of 74% for the parasite assemblage.

A total of 32 parasite individuals were collected: 15 in dry season and 17 in wet season. Parasites belonged to Platyhelminthes (Monogenea, Trematoda: Digenea) and Arthropoda (Crustacea: Copepoda). Copepods were the most represented group in terms of richness (5 spp., 55%) and abundance (18 individuals, 56%). Monogeneans accounted for 30% of total richness (3 spp.) and 31% of total abundance (9 individuals). Digenean trematodes were represented by a single species (10%) and four individuals (13%). In total, nine parasite species were identified (Table 2): *Allopyragraphorus caballeroi*, *Pseudomazocraes* sp., Microcotylidae sp., *Stephanostomum* sp., *Caligus robustus*, *Caligus sclerotinosus*, *Caligus* sp., *Caligus* sp.1, and *Lernanthropus giganteus*. It is important to note that none of the parasite species identified in *Caranx caballus* (Table 2) are zoonotic or of public health concern. Their significance lies in their ecological role as bioindicators of host diet, trophic interactions, and fisheries stock structure, rather than in sanitary risk to human consumers.

Species richness at the component community level did not differ between seasons (dry season: 6 spp.; wet season: 9 spp.; t = 0.73, *p* = 0.469). Prevalence comparisons showed no significant differences between seasons (χ^2^ = 0.011, *p* = 0.916; Fisher *p* = 1). Similarly, abundance did not differ between seasons (MW, U = 18.5, *p* = 0.83). The copepod *Caligus* sp.1 was the most dominant species (10 individuals, IBP = 0.31) and exhibited the highest prevalence across seasons (Table 3). Shannon–Wiener diversity was lower in dry season (H’ = 1.61) compared to wet season (H’ = 2.03), though the difference was not statistically significant (t = −1.62, *p* = 0.12).

Six parasite species (*A. caballeroi*, *Pseudomazocraes* sp., *C. robustus*, *C. sclerotinosus*, *Caligus* sp.1, *Stephanostomum* sp.) were present in both seasons. Microcotylidae sp., *L. giganteus*, and *Caligus* sp. were exclusive to dry season. Two immature copepods (*C. robustus*) were identified, one in each season, both at the chalimus stage (Table 2). Mean parasite richness per infected fish was 0.78 species (range 1–2) in dry season and 0.54 species (range 1–2) in wet season. Mean abundance per infected fish was 0.83 parasites (95% CI: 0.39–1.53; bootstrap 2000) in dry season and 0.57 parasites (95% CI: 0.29–0.82; bootstrap 2000) in wet season. Mean intensity in dry season was 1.88 parasites per infected fish (range 1–3; 95% CI: 1.25–2.38; bootstrap 1800). Brillouin diversity ranged from 0.597± 0.22 in dry season and 0.347 ± 0.11 in wet season, with higher average values in dry season, though differences were not significant (t = 1.93, *p* = 0.07).

No significant differences in prevalence were observed between males and females (χ^2^ = 0.032, *p* = 0.858). Overall prevalence was highest for *Caligus* sp.1 (31%), which was also the most abundant parasite in both seasons (five individuals per season), contributing to its designation as the species with the greatest specific importance. The monogenean *Pseudomazocraes* sp. had the highest prevalence among monogeneans (10.87%). The lowest prevalence values were recorded for Microcotylidae sp. and *L. giganteus* (3.1%). Qualitative similarity between seasons was 67% (Jaccard index). No correlation was found between prevalence and mean intensity across parasite species (rs = −0.5, *p* = 0.19).

### 3.3. Spatial Distribution of Parasite Assemblages

Spatial distribution analyses were applied to parasite species with prevalence greater than 8%, as this threshold ensured sufficient data for reliable aggregation testing while retaining all species with interpretable results. The analysis indicated that only *Allopyragraphorus caballeroi* exhibited a significantly aggregated distribution (*p* < 0.05). For other species, although some indices suggested aggregation, statistical tests did not confirm significance. *Caligus* sp.1 was identified as randomly distributed by IMC, and both dispersion and Morisita indices confirmed the absence of aggregation (Table 4).

Co-occurrence of parasite species was observed in four fish during dry season and three fish during wet season. Intraspecific co-occurrence occurred in two fish in dry season and one fish in wet season. The fish with the highest parasite load harbored three species simultaneously: *Pseudomazocraes* sp., *A. caballeroi*, and *Caligus* sp.1. Two fish presented co-occurrence patterns identical to *Caligus* sp.1 and *Stephanostomum* sp.

### 3.4. Diet Composition and Condition Factor

Stomach content analysis revealed six dietary categories: crustaceans (megalopae, mysids, stomatopods, isopods, amphipods, shrimps), fish larvae, salps, fish scales, polychaetes, and plant remains (Table 5). The overall vacuity index was 69.5%. Crustaceans were the most abundant prey group, particularly megalopae (early-stage brachyuran crabs).

Seasonal differences in diet composition were observed. During the dry season, mysids contributed 54.2% of crustacean abundance, followed by megalopa (39.6%). In the wet season, megalopa dominated (68%), while mysids accounted for only 4.3%. Across both seasons, megalopa were consistently the most important prey item (IRI season 1 = 39.58; IRI season 2 = 68.12), occurring in all stomachs with content (FA = 100), whereas mysids were present in only three stomachs (FA = 37.5).

Dietary diversity differed significantly between seasons (t = −3.38, *p* = 0.001), with higher diversity in wet season (H’ = 1.73) compared to season 1 (H’ = 1.23). Crustaceans were the only prey group with high relative importance (>60%), followed by fish scales and larvae, though at much lower percentages (Table 6).

**Figure 2:**
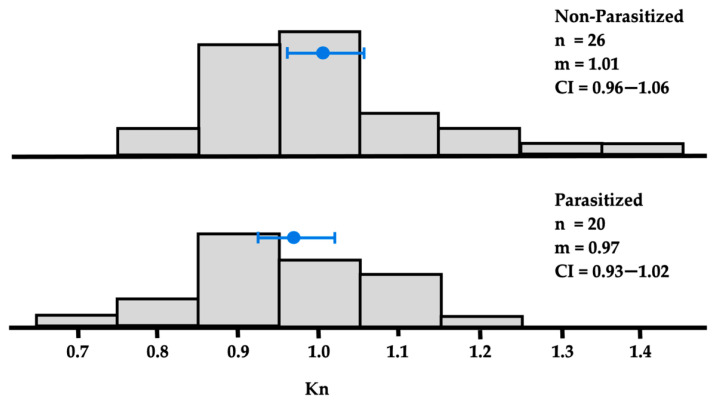
Relative condition factor (Kn) of non-parasitized and parasitized *Caranx caballus* in northern Chocó. n: number of fish examined; m: median (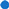), CI: 90% confidence interval of the median.

Analysis of the relative condition index (Kn) showed no significant differences between parasitized and non-parasitized fish (*p* = 0.177) (Figure 2). No associations were detected between condition factor and parasite presence, either overall or for the most prevalent species. This suggests that parasite infection did not measurably affect host condition within the sampled population, possibly due to low infection intensities or host resilience.

### 3.5. Environmental Records

**Figure 3:**
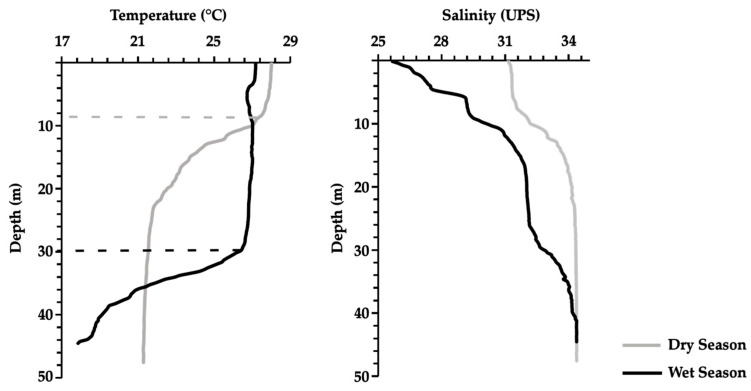
Vertical profiles of temperature and salinity in northern Chocó during the dry (January) and wet (April) seasons. The thermocline depth is indicated by a horizontal line, showing seasonal differences in water column structure.

Oceanographic monitoring revealed clear seasonal contrasts in surface conditions. During the dry season (January), mean sea surface temperature and salinity were higher than in the wet season (April) (Table 7). Vertical profiles further indicated a shallow thermocline in January, while in April, the thermocline deepened markedly (Figure 3).

### 3.6. Integrated Results: Parasite Assemblages, Host Diet, and Oceanographic Variability

Parasite assemblages in *C. caballus* were characterized by low richness and moderate prevalence, dominated by copepods, particularly *Caligus* sp.1, which exhibited the highest prevalence and abundance. *Allopyragraphorus caballeroi* displayed an aggregated distribution pattern. Diet analysis showed a strong reliance on crustaceans, especially megalopa, with seasonal variation in prey diversity. Host condition factors did not differ between parasitized and non-parasitized fish. Seasonal variability in oceanographic conditions was observed. During the wet season, deeper mixing enhanced oxygen concentrations in the water column and coincided with greater dietary diversity in *C. caballus*. In contrast, the dry season was characterized by warmer, saltier surface waters and a shallow thermocline, conditions associated with reduced prey diversity and slightly higher parasite richness.

## 4. Discussion

This study represents the first systematic characterization of the metazoan parasite community associated with *Caranx caballus* in the northern Colombian Pacific, a region of high artisanal fishing activity but limited parasitological research. A total of 46 individuals were collected (18 during the dry season and 28 during the wet season), and we acknowledge that this seasonal imbalance constrains the robustness of statistical comparisons. Moreover, although our sampling effort achieved 74% representativity, the absence of a formal power analysis, due to logistical constraints inherent to artisanal fisheries, further limits statistical inference. Accordingly, our results should be interpreted as baseline information that provides a foundation for future expanded sampling. Despite these constraints, the dataset contributes novel baseline knowledge on parasite assemblages in *C. caballus* and situates local artisanal fisheries within the broader Eastern Tropical Pacific (ETP) framework, where even modest datasets have proven valuable for understanding parasite diversity, stock discrimination, and ecosystem monitoring under climate variability [13,19,21,56,57,58].

The 74% representativity indicates that the effort was sufficient to capture most of the parasite diversity present in *C. caballus*, although additional sampling would likely reveal further species and increase richness estimates, as reported in other tropical marine fish studies [7,13,59]. Overall, the parasite assemblage exhibited low richness and moderate prevalence, dominated by copepods—particularly *Caligus* sp.1—which emerged as the most abundant and prevalent species across seasons (Table 2 and Table 3). The detection of *Allopyragraphorus caballeroi* with aggregated distribution patterns (Table 4) underscores the heterogeneity of host–parasite interactions within this fishery. Importantly, these findings contribute novel baseline data for the northern Colombian Pacific and situate local artisanal fisheries within the broader ETP framework, where parasites are increasingly recognized as bioindicators of stock structure, ecosystem variability, and resilience under climate change [13,16,57,58,60].

Despite significant differences in host diet composition and environmental conditions between sampling periods (Table 5, Table 6 and Table 7), parasite communities exhibited seasonal stability in richness and prevalence. The strong reliance of *C. caballus* on crustacean prey, especially brachyuran megalopa, suggests trophic transmission pathways that may explain the dominance of copepod parasites. Infection intensities were generally low, and host condition factors did not differ between parasitized and non-parasitized individuals (Figure 2), indicating resilience of *C. caballus* under current parasitic pressures [58,59,60,61].

**Figure 4:**
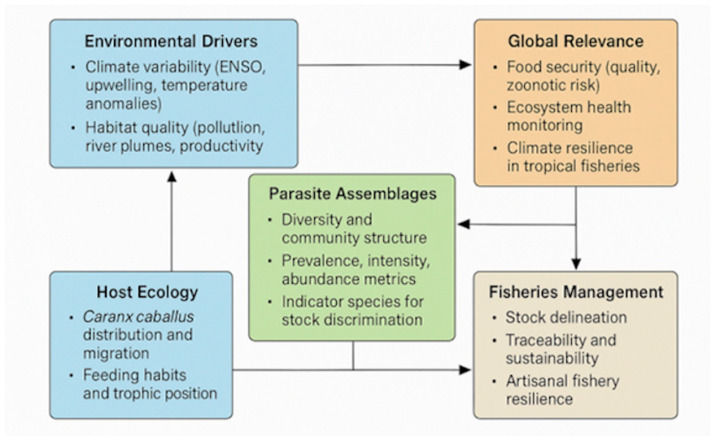
Conceptual framework linking parasite assemblages, host ecology, and climate drivers in artisanal fisheries. Parasites are positioned as dual indicators: (1) biological markers for stock discrimination and fisheries management, and (2) sentinels of ecosystem change under climate variability, emphasizing implications for sustainability, food security, and climate resilience.

These findings underscore the dual role of parasites as ecological indicators. Locally, they provide insights into host feeding ecology, stock discrimination, and transmission dynamics. Globally, they serve as sentinels of ecosystem change under climate variability. By linking parasite assemblages to diet, host condition, and oceanographic drivers (Figure 4), this study situates artisanal fisheries of the Colombian Pacific within broader debates on sustainability, food security, and resilience in tropical marine ecosystems.

Of the 30 parasite species previously reported for *C. caballus*, we identified three (10%) in our samples. Notably, three copepods (*Caligus sclerotinosus*, *Caligus* sp., *Caligus* sp.1), two monogeneans (Microcotylidae sp. and *Pseudomazocraes* sp.), and one digenean (*Stephanostomum* sp.) constitute new records. All species reported here are the first for Colombia, thereby extending their known distribution within the Eastern Tropical Pacific (ETP).

Host condition was evaluated using Fulton’s condition factor, derived from length–weight relationships [49,50], and compared between parasitized and non-parasitized individuals. Although no significant differences were detected, this standardized approach provides a transparent baseline for assessing parasite influence on host health. Contrary to expectations from previous studies, no significant relationships were found between host size, sex, and parasite prevalence. Similar results were reported by Violante-González et al. [13] in Mexico, where total length was unrelated to prevalence in two of three localities. The absence of such associations in our study may reflect the predominance of immature individuals, whose habitat use and resource exploitation differ from adults [62,63,64]. Juvenile habitat partitioning can influence parasite exposure, as infracommunities and component communities are shaped by host resource use and trophic position [12]. Given that prevalence and mean intensity were not linked to size, we suggest that fish across sampled sites and seasons did not exhibit significant differences in resource use, consistent with the >50% similarity in parasite composition between seasons.

Parasite communities at both infracommunity and component levels exhibited low richness, low diversity, and dominance by a single species. The categorization of low richness in the parasitofauna of *C. caballus* from Colombia is relative and based on comparisons with published studies of carangid populations in the Mexican Pacific [17,56], which reported higher species counts and prevalence under similar sampling conditions. These benchmarks justify our classification of the assemblage as low richness, reflecting both the limited number of species detected and the dominance of a single taxonomic group. Component community richness did not vary significantly between seasons, differing from records for other carangids [5,14]. The addition of new species in season 2 likely reflects increased sample size rather than structural change, consistent with nested subset patterns described by Guegán & Hugueny [65]. Such nestedness may arise from environmental influences on free-living infective stages or host distribution, leading to parasite gains or losses [5,66].

Comparisons with Mexican populations of *C. caballus* [13] revealed lower prevalence and mean intensity in Colombian fish, despite shared species (*A. caballeroi*, *C. robustus*). Parasite associations are rarely stable across host populations as communities are dynamic assemblages shaped by climate and latitude [6]. Interestingly, our results contrast with [7], who reported increased ectoparasite prevalence at lower latitudes, suggesting that local environmental conditions and host ecology may override latitudinal trends.

Copepods were the most represented group (56%), consistent with findings in Mexico [13]. As one of the most diverse groups of marine fish ectoparasites, copepods, together with monogeneans, dominate parasite assemblages globally [8]. Their prevalence in our study aligns with host diet, which was dominated by crustaceans, particularly brachyuran megalopa. This result is consistent with Saucedo-Lozano et al. [35], although their January samples were dominated by cnidarians and mollusks, highlighting regional dietary variability. The predominance of crustaceans in both seasons suggests a relatively homogeneous prey supply, corroborated by Espinal-García et al. [67], who reported year-round availability of brachyuran larvae in Colombian Pacific waters. Such resource homogeneity may limit exposure to intermediate hosts of endoparasites, explaining the absence of nematodes, acanthocephalans, and other taxa commonly reported in Mexican populations where diets included penaeid shrimps and adult fishes. The consumption of larval fishes in our samples may further explain the absence of nematodes, which typically infect intermediate hosts at later developmental stages [68].

The strong reliance of *C. caballus* on crustacean prey, especially brachyuran megalopa, suggests trophic transmission pathways that may explain the dominance of copepod parasites. Copepods typically have direct life cycles, attaching directly to hosts without intermediate hosts [69,70]. However, frequent ingestion of free-living crustaceans such as mysids and megalopa may increase exposure opportunities through shared habitats and trophic overlap, reinforcing the ecological link between host diet and parasite prevalence [57,59,71]. This mechanism highlights how host feeding behavior directly shapes parasite community structure, consistent with reports in other tropical marine fishes where crustacean-rich diets are associated with higher copepod infection rates [58,61,72]. Such findings align with broader evidence that caligid copepods exploit trophic pathways opportunistically, reflecting their ecological versatility and capacity to dominate parasite assemblages in crustacean-dependent fish population [56,60].

Spatial distribution analyses revealed contrasting patterns: *A. caballeroi* exhibited aggregated distribution, which is consistent with monogeneans that remain on hosts and rapidly form dense populations [73]. In contrast, *Caligus* sp.1 displayed random distribution, likely reflecting its mobility and colonization strategy. Caligid copepods possess swimming appendages that enable dispersal across host populations, reducing the need for aggregation and minimizing intraspecific competition. Their presence in plankton samples supports this interpretation, highlighting their capacity to exploit hosts opportunistically across heterogeneous environments [15].

Host-genus-specialist parasites often dominate communities, while most parasites behave as generalists [74]. In this study, the dominant species was a copepod, a group generally recognized as taxonomic generalists capable of parasitizing diverse fish families. For example, *Caligus robustus* has been reported in scombrids, lutjanids, and haemulids [75]. The absence of central specialist species in our samples limits the predictability of parasite community structure in *C. caballus*, a conclusion supported by the lack of correlation between prevalence and mean intensity. Beyond these structural patterns, parasites fulfill a dual role: they function as biological tags for stock discrimination [13,14,15,21,56] and act as sentinels of ecosystem change under climate variability, with shifts in prevalence and assemblage composition reflecting broader environmental and trophic alterations [4,5,6,7,8,16,17,18,66].

Environmental variation, particularly in temperature and salinity between seasons, may have influenced parasite diversity, even though overall community structure appeared temporally stable (Table 7). These shifts in oceanographic structure are ecologically relevant. Warmer, saltier, and more stratified waters in the dry season may favor parasite transmission by enhancing host aggregation near the surface, whereas cooler, fresher, and more mixed conditions in the wet season could reduce parasite encounter rates and alter host feeding opportunities. Among monogeneans, *A. caballeroi* has been reported from Peru in *Caranx hippos* (likely misidentified, as this species does not occur in the Pacific, suggesting *C. caninus* instead; FishBase, accessed 2 February 2025). This indicates a broad distribution across the ETP. Similarly, the genus *Pseudomazocraes* (e.g., *P. selene*) has been reported in the Atlantic parasitizing *Selene vomer*. These records challenge the expectation that monogeneans are strictly host-genus specialists [76,77]. In our study, the unidentified microcotylid and *Pseudomazocraes* sp. may represent host-specific parasites of *C. caballus*, but further taxonomic resolution is required.

The only endoparasite recorded was the digenean *Stephanostomum* sp., typically found in the digestive tract, especially the intestine [78,79]. In this case, individuals were located in the esophagus, in a nearly external position. Their presence provides insights into the trophic role of *C. caballus*, as digeneans require intermediate hosts to complete their life cycle. However, species-level identification and life-cycle studies are necessary to determine whether *C. caballus* functions as a secondary intermediate host or as a definitive host in the region.

Taken together, the patterns observed in parasite prevalence, diversity, and aggregation, along with the associations between host condition and diet composition, underscore the multifaceted role of parasites in artisanal fisheries. These findings highlight how parasite assemblages not only reflect local ecological processes—such as trophic transmission and seasonal variability—but also provide signals of broader ecosystem dynamics influenced by climate drivers.

## 5. Conclusions

In the northern Colombian Pacific, parasite assemblages of *Caranx caballus* were characterized by low richness, moderate prevalence, and dominance by copepods, particularly species of *Caligus*. The absence of specialist taxa and the predominance of generalist parasites emphasize the dynamic and opportunistic nature of parasite communities in artisanal fisheries. Although infection intensities were low and host condition remained unaffected, ecological patterns—such as the aggregated distribution of *Allopyragraphorus caballeroi* and the trophic linkages between crustacean prey and copepod parasites—underscore the integrative role of parasites in shaping host ecology and reflecting environmental variability.

From a management perspective, parasites provide valuable insights into host population structure, trophic interactions, and ecosystem health. Their integration into fisheries monitoring frameworks could enhance sustainability assessments and guide adaptive strategies for artisanal fisheries, particularly in regions vulnerable to climate-driven oceanographic change. Future research should expand sample sizes, incorporate longitudinal datasets, and examine parasite life cycles in greater detail to clarify host roles and transmission pathways. Comparative studies across the Eastern Tropical Pacific would further contextualize findings from the Colombian Pacific within broader regional patterns, thereby strengthening the global relevance of parasite ecology in fisheries science.

Parasite studies thus offer a powerful lens into the complex interactions among hosts, prey, and environmental drivers, underscoring their potential as ecological sentinels in the pursuit of sustainable fisheries and resilient coastal communities. Although temperature and salinity profiles were described to contextualize seasonal oceanographic conditions, no statistical associations with parasite infection levels were tested in this study. Future research should address this link to better understand the role of environmental variability—such as thermocline depth and water column structure—in shaping host–parasite interactions in tropical upwelling ecosystems. Similarly, histopathological evaluation was not included here; subsequent studies could incorporate tissue-level analyses to assess the pathological impacts of parasite infections on host organs, thereby complementing the ecological and epidemiological perspectives presented. Despite these limitations, our findings expand the known distribution of several parasite species within the Eastern Tropical Pacific and provide baseline information for the Colombian Pacific, a region where parasitological data remain scarce yet ecologically significant.

## Figures and Tables

**Table 1:** Morphometric and biological information for *Caranx caballus* captured in the northern Colombian Pacific during two seasons. Data include size, weight, sex, and infection status of fish examined for parasitological analysis. ND = not determine. Mean ± Standard Deviation.

Attribute	Dry Season	Wet Season
N	18	28
Mean Total Length (mm)	354.72 ± 27.3	252.88 ± 10.87
Mean Weight (g)	500 ± 78	311.8 ± 60
Males	11	ND
Females	6	ND
Not Sexed	1	28
Infected	8	12

**Table 2:** Composition of the metazoan parasite community of *Caranx caballus* in the northern Colombian Pacific. N = number of parasite individuals; In = number of infected fish. Values summarize taxonomic groups and infection occurrence across hosts.

Taxon	Dry Season		Wet Season		Total	
	N	In	N	In	N	In
Monogenea						
Microcotylidae sp.	-	-	1	1	1	1
*Pseudomazocraes* sp.	4	3	2	1	6	4
*Allopyragrophorus caballeroi*	1	1	2	1	3	2
						
Digenea						
*Stephanostomum* sp.	2	2	2	2	4	4
						
Copepoda						
*Caligus robustus*	2	2	2	2	4	4
*Caligus sclerotinosus*	1	1	1	1	2	2
*Caligus* sp.	-	-	1	1	1	1
*Caligus* sp.1	5	5	5	4	10	9
*Lernanthropus giganteus*	-	-	1	1	1	1

**Table 3:** Epidemiological index of *Caranx caballus* by season and overall (Total). n = number of infected fish; N = number of parasites; P = prevalence (%); MA = mean abundance; MI = mean intensity; SI = species importance index. “General” includes all parasites pooled across taxa. N/A = not applicable.

	N	N	P	MA	MI	Range	SI
Taxon	Dry Season						
*Pseudomazocraes* sp.	3	4	16.67	0.22	1.33	1	38.89
*Allopyragrophorus caballeroi*	1	1	5.56	0.06	1	1	11.11
*Stephanostomum* sp.	2	2	11.11	0.11	1	1	22.22
*Caligus robustus*	2	2	11.11	0.11	1	1	11.11
*Caligus sclerotinosus*	1	1	5.56	0.06	1	1	11.11
*Caligus* sp.1	5	5	27.78	0.28	1	1	55.56
Taxon	Wet Season						
Microcotylidae sp.	1	1	3.57	0.04	1	1	7.14
*Pseudomazocraes* sp.	2	2	7.10	0.07	1	1	7.14
*Allopyragrophorus caballeroi*	1	2	3.57	0.07	2	1–2	10.71
*Stephanostomum* sp.	2	2	7.14	0.07	1	1	14.29
*Caligus robustus*	2	2	7.10	0.07	1.25	1	21.43
*Caligus sclerotinosus*	1	1	3.57	0.04	1	1	7.14
*Caligus* sp.	1	1	3.57	0.04	1	1	7.14
*Caligus* sp.1	4	5	14.29	0.18	1	1–2	32.14
*Lernanthropus giganteus*	1	1	3.57	0.04	1	1	7.14
Taxon	Total						
General	29	32	41.30	0.70	1.68	1–4	N/A
Microcotylidae sp.	1	1	2.17	0.02	1	1	4.35
*Pseudomazocraes* sp.	4	6	10.87	0.13	0.83	1–2	23.91
*Allopyragrophorus caballeroi*	2	3	4.35	0.07	0.67	1–2	10.87
*Stephanostomum* sp.	3	4	6.52	0.09	0.75	1–2	15.22
*Caligus robustus*	4	4	8.70	0.09	1	1	13.04
*Caligus sclerotinosus*	2	2	4.35	0.04	1	1	8.70
*Caligus* sp.	1	1	2.17	0.02	1	1	4.35
*Caligus* sp.1	9	10	17.39	0.22	0.8	1–2	39.13
*Lernanthropus giganteus*	1	1	2.17	0.02	1	1	4.35

**Table 4:** Spatial aggregation indices of the most prevalent parasites of *Caranx caballus*. S1 = *Pseudomazocraes* sp.; S2 = *Allopyragraphorus caballeroi*; S3 = *Stephanostomum* sp.; S4 = *Caligus robustus*; S5 = *Caligus* sp.1. MA = mean abundance. *p* = significance value of K index (ID) and Morisita’s Im test.

**Index**	**S1**	**S2**	**S3**	**S4**	**S5**	**General**
K (ID)	1.23	1.64	0.93	0.91	1.27	1.21
*p*	0.14	0.01 *	0.6	0.64	0.16	0.16
IG	0.01	0.01	0.00	0.00	0.01	3.38
IMC	0.36	0.7	0.03	0.02	0.22	0.9
(MA)	0.13	0.07	0.09	0.09	0.22	0.7
Im	3.07	15.33	0.00	0.00	2.80	1.3

Spatial aggregation: Uniform (u), Conglomerate (c), Aleatory (a). K: <1 = u, >1 = c, =1 = a; ICS: <0 = u, >0 = c, =0 = a; IG: <0 = u, >0 = c, =0 = a; IMC: <MA = u, >MA = c, = MA = a; Im: <1 = u, >1 = c, =1 = a; * Statistically significant values.

**Table 5:** Seasonal diet composition of *Caranx caballus* in northern Chocó. N = number of individuals per prey category; Fr = relative frequency (%). Values indicate seasonal variation in prey categories consumed.

Item	Dry Season		Wet Season		General	
	N	Fr (%)	N	Fr (%)	N	Fr (%)
Crustaceans	48	90	138	62	186	68.6
Salps	2	4	10	4.5	12	4.4
Fish scales	1	2	38	17	39	14.4
Polichaeta	-	-	5	2.2	5	1.8
Fish larvae	1	2	27	12.1	28	10.3
Plant remains	1	2	-	-	1	0.4

**Table 6:** Importance of dietary items observed in *Caranx caballus* from northern Chocó. Fr = frequency (number of stomachs containing the item); FO = frequency of occurrence (%); N = total abundance; NF = numerical frequency (%); IRI = index of relative importance. Values indicate the relative contribution of each prey category to overall diet composition.

Item	Fr	FO	N	NF	IRI	Importance
Crustaceans	30	93.75	186	68.63	64.35	High
Salps	7	21.87	12	4.43	0.97	Low
Fish scales	12	37.5	39	14.39	5.40	Low
Polichaeta	1	3.12	1	0.37	0.01	Low
Fish larvae	16	50	28	10.33	5.17	Low
Plant remains	4	12.5	5	1.85	0.23	Low

**Table 7:** Seasonal comparison of oceanographic variables (surface temperature [T], salinity [S]. and thermocline depth [D_T_]) in northern Chocó. Values are expressed as mean ± standard deviation. Dry season (January); wet season (April); Z: Z adjusted from Mann–Whitney U test statistic; *p*: *p*-value.

Variable	Dry Season	Wet Season	Z	*p*
T	28.3 (±0.5)	27.0 (±0.3)	1.97	0.049
S	31.1 (±0.2)	26.2 (±0.5)	2.29	0.048
D_T_	8.5	30		

## Data Availability

Data supporting the findings of this study are available within the article; additional data are available from the corresponding author upon reasonable request.

## References

[1] Poulin R., Mouritsen K.N. (2006). Climate change and parasitism in intertidal systems. J. Helminthol..

[2] Klimpel S., Kuhn T., Münster J., Dörge D.D., Klapper R., Kochmann J. (2019). Parasites of Marine Fish and Cephalopods.

[3] Bateman K.S., Feist S.W., Bignell J.P., Bass D., Behringer D.C., Silliman B.R., Lafferty K.D. (2020). Marine pathogen diversity. Marine Disease Ecology.

[4] Byers J.E. (2021). Marine parasites and disease in the era of global climate change. Annu. Rev. Mar. Sci..

[5] Schade F.M., Raupach M.J., Wegner K.M. (2016). Seasonal variation in parasite infection patterns of marine fish species from the Northern Wadden Sea in relation to interannual temperature fluctuations. J. Sea Res..

[6] Poulin R. (2007). General laws in parasite ecology?. Parasitology.

[7] Rohde K., Heap M. (1998). Latitudinal differences in parasite richness and structure. Int. J. Parasitol..

[8] Rohde K. (2005). Marine Parasitology.

[9] McLaughlin J.P., Morton D.N., Lafferty K.D., Behringer D.C., Silliman B.R., Lafferty K.D. (2020). Parasites in marine food webs. Marine Disease Ecology.

[10] Behringer D.C., Wood C.L., Krkošek M., Bushek D., Behringer D.C., Silliman B.R., Lafferty K.D. (2020). Disease in fisheries and aquaculture. Marine Disease Ecology.

[11] Marcogliese D.J. (2002). Food webs and the transmission of parasites to marine fish. Parasitology.

[12] Lafferty K.D. (2013). Parasites in marine food webs. Bull. Mar. Sci..

[13] Violante-González J., Gallegos-Navarro Y., Monks S., García-Ibánez S., Rojas-Herrera A.A., Pulido-Flores G., Villeiras-Salinas S., Larumbe-Morán E. (2016). Parasites of the green jack *Caranx caballus* (Pisces: Carangidae) in three locations from Pacific coasts of Mexico, and their utility as biological tags. Rev. Mex. Biodivers..

[14] Boada M., Bashirullah A., Alió J., Ortíz L. (2015). Distribución espacial y descriptores comunitarios de ectoparásitos en branquias de *Caranx ruber*. Saber.

[15] Grutter A.S. (1998). Habitat-related differences in parasite abundance indicate fish movement. J. Fish Biol..

[16] Nachev M., Sures B. (2016). Environmental parasitology: Parasites as accumulation bioindicators in the marine environment. J. Sea Res..

[17] Sures B., Nachev M., Schwelm J., Grabner D., Selbach C. (2023). Environmental parasitology: Stressor effects on aquatic parasites. Trends Parasitol..

[18] Byers J.E. (2020). Effects of climate change on parasites and disease in estuarine and nearshore environments. PLoS Biol..

[19] Perdiguero-Alonso D., Montero F.E., Kostadinova A., Raga J.A., Barrett J. (2008). Random forests using parasites as biological tags. Int. J. Parasitol..

[20] Yamaguchi S., Sawada K., Yusa Y., Iwasa Y. (2013). Dwarf males, large hermaphrodites and females in marine species: A dynamic optimization model of sex allocation and growth. Theor. Popul. Biol..

[21] Timi J.T., MacKenzie K. (2015). Parasites in fisheries and mariculture. Parasitology.

[22] Timi J.T., Buchmann K. (2023). A century of parasitology in fisheries and aquaculture. J. Helminthol..

[23] Timi J.T., Rossin M.A., Alarcos A.J., Braicovich P.E., Cantatore D.M.P., Lanfranchi A.L. (2011). Fish trophic level and larval parasite assemblages. Int. J. Parasitol..

[24] Poulin R., Leung T.L.F. (2011). Body size, trophic level, and parasite transmission routes. Oecologia.

[25] Bennett J., Presswell B., Poulin R. (2023). Tracking life cycles of parasites across a broad taxonomic scale in a marine ecosystem. Int. J. Parasitol..

[26] Bennett J., Poulin R., Presswell B. (2022). Large-scale genetic investigation of nematode diversity and their phylogenetic patterns in New Zealand’s marine animals. Parasitology.

[27] Van Steenkiste N.W., Wakeman K.C., Söderström B., Leander B.S. (2023). Patterns of host–parasite associations between marine meiofaunal flatworms (Platyhelminthes) and rhytidocystids (Apicomplexa). Sci. Rep..

[28] Walther G.R., Post E., Convey P., Menzel A., Parmesan C., Beebee T.J., Fromentin J.M., Bairlein F. (2002). Ecological responses to recent climate change. Nature.

[29] Stenseth N.C., Mysterud A., Ottersen G., Hurrell J.W., Chan K.S., Lima M. (2002). Ecological effects of climate fluctuations. Science.

[30] Marcogliese D.J., Gendron A.D., Plante C., Fournier M., Cyr D. (2006). Parasites of spottail shiners (*Notropis hudsonius*) in the St. Lawrence River: Effects of municipal effluents and habitat. Can. J. Zool..

[31] Shea J., Kersten G.J., Puccia C.M., Stanton A.T., Stiso S.N., Helgeson E.S., Back E.J. (2012). The use of parasites as indicators of ecosystem health as compared to insects in freshwater lakes of the Inland Northwest. Ecol. Indic..

[32] SQUALUS (2008). Pesquería artesanal de la zona norte del Pacífico colombiano.

[33] Díaz J.M., Guillot L., Velandia M.C. (2016). La pesca artesanal en la costa norte del Pacífico colombiano: Un horizonte ambivalente.

[34] Mair J.M., Cipriani R., Guzmán H.M., Usan D. (2012). Fishery of *Caranx caballus* in Las Perlas Archipelago. Rev. Biol. Trop..

[35] Saucedo-Lozano M., Bernal-Ornelas I.H., Espino-Barr E., Garcia-Boa A., Cabral-Solís E.G., Puente-Gómez M. (2012). Feeding habits of *Caranx caballus* in Manzanillo, Mexico. Open Mar. Biol. J..

[36] Giraldo A., Valencia B., Martínez T., Ramírez D. (2008). Condiciones oceanográficas en Punta Cruces y Cabo Marzo. Chocó, Paraíso por Naturaleza.

[37] Vidal-Martínez V.M., Aguirre-Macedo M.L., Scholz T., González-Solís D., Mendoza-Franco É.F. (2002). Atlas de helmintos parásitos de cíclidos de México.

[38] Boxshall G.A., Halsey S.H. (2004). An Introduction to Copepod Diversity.

[39] Bray R.A., Cribb T.H. (2003). Species of *Stephanostomum* from Australian and South Pacific fishes, including five new species. Syst. Parasitol..

[40] Ho J.-S., Gómez S., Ogawa K., Aritaki M. (2004). Two Caligidae species new to Japan. Syst. Parasitol..

[41] Bush A.O., Lafferty K.D., Lotz J.M., Shostak A.W. (1997). Parasitology meets ecology on its own terms: Margolis et al. revisited. J. Parasitol..

[42] Giraldo A., Valencia B., Acevedo J.D., Rivera M. (2011). Protocolo para la obtención de datos oceanográficos y de plancton.

[43] Cressey R., Cressey B. (1980). Parasitic copepods of Mackerel- and Tuna like fishes (Scombridae) of the world. Smithson. Contrib. Zool..

[44] Thatcher V. (1993). Trematódeos Neotropicais.

[45] Hayes P., Justine J.-L., Boxshall G. (2012). The genus *Caligus*: Two new species and nomenclatural notes. Zootaxa.

[46] Bursey C.R., Goldberg S.R., Parmelee J.R. (2001). Gastrointestinal helminths of anurans from Reserva Cuzco Amazónico, Peru. Comp. Parasitol..

[47] Iannacone J., Sánchez V., Olazábal N., Salvador C., Alvariño L., Molano J. (2012). Ecological indices of parasites of *Scartichthys gigas* (Steindachner, 1876) (Perciformes: Blenniidae) of the coasts of Lima, Peru. Neotrop. Helminthol..

[48] Bush A., Holmes J. (1986). Intestinal parasites of lesser scaup ducks: Patterns of association. Can. J. Zool..

[49] Froese R. (2006). Cube law, condition factor and weight–length relationships: History, meta-analysis and recommendations. J. Appl. Ichthyol..

[50] Jin S., Yan X., Zhang H., Fan W. (2015). Weight–length relationships and Fulton’s condition factors of skipjack tuna (*Katsuwonus pelamis*) in the western and central Pacific Ocean. PeerJ.

[51] Windell J.T., Ricker W.E. (1971). Food analysis and digestion rate. Methods for Assessment of Fish Production in Fresh Waters.

[52] Silva M., Stuardo J. (1985). Demersal fish feeding and benthos in Bahía Coliumo. Gayana Zool..

[53] Yáñez-Arancibia A., Curiel-Gómez J., Leyton V. (1976). Ecology of *Galeichthys caerulescens* in Guerrero lagoon system. An. Cent. Cienc. Mar Limnol..

[54] Rózsa L., Reiczigel J., Majoros G. (2000). Quantifying parasites in host samples. J. Parasitol..

[55] Rosenberg M.S., Anderson C.D. (2011). PASSaGE2: Pattern analysis and spatial statistics. Methods Ecol. Evol..

[56] Violante-González J., Monks S., Gallegos-Navarro Y., Santos-Bustos N.G., Villalba-Vasquez P.J., Miranda-Delgado J.E., Carpio-Hernández D.I. (2019). Metazoan parasite communities of the Pacific jack *Caranx caninus* (Pisces: Carangidae): Exploring the variability of their parasite communities. J. Nat. Hist..

[57] Jacobson K.C., Marcogliese D.J., MacKenzie K. (2024). Parasites of small pelagics reflect their role in marine ecosystems. Mar. Ecol. Prog. Ser..

[58] Villalba-Vasquez P.J., Violante-González J., Pulido-Flores G., Monks S., Rojas-Herrera A.A., Flores-Rodríguez P., Cayetano C.V., Rosas-Acevedo J.L., Santos-Bustos N.G. (2022). Metazoan parasite communities of the Pacific red snapper, *Lutjanus peru* (Perciformes: Lutjanidae): Interannual variations in parasite communities. J. Helminthol..

[59] Koepper S., Nuryati S., Palm H.W., Wild C., Yulianto I., Kleinertz S. (2022). Metazoan endoparasite fauna and feeding ecology of commercial fishes from Java, Indonesia. Parasitol. Res..

[60] Miranda-Delgado J.E., Violante-González J., Monks S., Rojas-Herrera A.A., García-Ibáñez S., Flores-Rodríguez P., Romero-Ramírez Y., Santos-Bustos N.G. (2019). Factors linked to interannual variation in the metazoan parasite communities of black skipjack, Euthynnus lineatus (Pisces: Scombridae). Invertebr. Biol..

[61] Woodstock M.S., Blanar C.A., Sutton T.T. (2020). Diet and parasites of a mesopelagic fish assemblage in the Gulf of Mexico. Mar. Biol..

[62] Hughes N., Grand T. (2000). Predicting size-structured fish distributions across temperature gradients. Environ. Biol. Fishes.

[63] Stoner A.W. (2004). Environmental effects on fish feeding ecology. J. Fish Biol..

[64] Helfman G., Collette B., Facey D., Bowen B. (2009). The Diversity of Fishes.

[65] Guegán J.F., Hugueny B. (1994). Nested parasite species subsets in tropical fish. Oecologia.

[66] Timi J.T., Poulin R. (2003). Repeatability of parasite community structure across host populations. Int. J. Parasitol..

[67] Espinal-García P., Giraldo A., Londoño-Mesa M., Mejía-Ladino L.M. (2012). Variabilidad de larvas de crustáceos y poliquetos en Bahía Málaga. Boletín Investig. Mar. Costeras.

[68] Anderson R.C. (2000). Nematode Parasites of Vertebrates: Their Development and Transmission.

[69] Ohtsuka S., Madinabeitia I., Yamashita H., Maran B.V., Suárez-Morales E., Ho J.S. (2018). Planktonic phases in symbiotic copepods: A review. Bull. South. Calif. Acad. Sci..

[70] Bass D., Rueckert S., Stern R., Cleary A.C., Taylor J.D., Ward G.M., Huys R. (2021). Parasites, pathogens, and other symbionts of copepods. Trends Parasitol..

[71] Locke S.A., Marcogliese D.J., Tellervo Valtonen E. (2014). Vulnerability and diet breadth predict larval and adult parasite diversity in fish of the Bothnian Bay. Oecologia.

[72] Ferré-Alcántara K., Minaya D., Alvariño L., Iannacone J. (2023). Ectoparasitic community of the gills of Pacific sierra *Scomberomorus sierra* Jordan & Starks, 1895 (Actinopteri: Scombridae) from northern Peru. Rev. Mus. Argent. Cienc. Nat..

[73] Sikkel P.C., Welicky R.L., Smit N., Bruce N., Hadfield K. (2019). The Ecological Significance of Parasitic Crustaceans. Parasitic Crustacea.

[74] Suárez-Morales E., Camisotti H., Martín A. (2012). A new *Caligus* species from Venezuelan plankton. ZooKeys.

[75] Kazachenko V.N., Kovaleva N.N., Ha Duy Ngo Van Ha N., Thanh N.V. (2014). Redescription of three caligid species of the genus *Caligus* Müller, 1785 (Copepoda: Caligidae), parasites of marine fish *Decapterus* sp. (Perciformes: Carangidae) from Tonkin Gulf, Vietnam. Tap Chi Sinh Hoc.

[76] Poulin R. (1992). Determinants of host specificity. Int. J. Parasitol..

[77] Whittington I.D., Cribb B.W., Hamwood T.E., Halliday J.A. (2000). Host-specificity of monogenean (Platyhelminth) parasites: A role for anterior adhesive areas?. Int. J. Parasitol..

[78] Pérez-Ponce de León G., León-Règagnon V., Scott M. (1998). *Theletrum lamothei* sp. nov. (Digenea), parasite of *Echidna nocturna* from Cuajiniquil, Guanacaste, and other digenes of marine fishes from Costa Rica. Rev. Biol. Trop..

[79] Lozano C., Ubeda J.M., De Rojas M., Ariza C., Guevara D.C. (2001). Estudio de digénidos de peces marinos del sur de la Península Ibérica. Rev. Ibérica Parasitol..

